# *In silico* and *in vivo* evaluation of the anti-cryptosporidial activity of eugenol

**DOI:** 10.3389/fvets.2024.1374116

**Published:** 2024-03-07

**Authors:** Hattan S. Gattan, Majed H. Wakid, Rowaid M. Qahwaji, Sarah Altwaim, Haifaa A. Mahjoub, Mashael S. Alfaifi, Hayam Elshazly, Wafa Abdullah I. Al-Megrin, Eman Abdullah Alshehri, Hatem A. Elshabrawy, Asmaa M. El-kady

**Affiliations:** ^1^Department of Medical Laboratory Sciences, Faculty of Applied Medical Sciences, King Abdulaziz University, Jeddah, Saudi Arabia; ^2^Special Infectious Agents Unit, King Fahd Medical Research Center, Jeddah, Saudi Arabia; ^3^Department of Clinical Microbiology and Immunology, Faculty of Medicine, King Abdulaziz University, Jeddah, Saudi Arabia; ^4^Biological Sciences Department, College of Sciences and Arts, King Abdulaziz University, Rabigh, Saudi Arabia; ^5^Department of Epidemiology, Faculty of Public Health and Health Informatics, Umm Al-Qura University, Mecca, Saudi Arabia; ^6^Department of Biology, Faculty of Sciences-Scientific Departments, Qassim University, Buraidah, Saudi Arabia; ^7^Department of Zoology, Faculty of Science, Beni-Suef University, Beni Suef, Egypt; ^8^Department of Biology, College of Science, Princess Nourah Bint Abdulrahman University, Riyadh, Saudi Arabia; ^9^Department of Zoology, College of Science, King Saud University, Riyadh, Saudi Arabia; ^10^Department of Molecular and Cellular Biology, College of Osteopathic Medicine, Sam Houston State University, Conroe, TX, United States; ^11^Department of Medical Parasitology, Faculty of Medicine, South Valley University, Qena, Egypt

**Keywords:** *Cryptosporidium*, immunocompromised, eugenol, nitazoxanide, iNOS

## Abstract

**Background:**

Cryptosporidiosis is an opportunistic parasitic disease widely distributed worldwide. Although *Cryptosporidium* sp. causes asymptomatic infection in healthy people, it may lead to severe illness in immunocompromised individuals. Limited effective therapeutic alternatives are available against cryptosporidiosis in this category of patients. So, there is an urgent need for therapeutic alternatives for cryptosporidiosis. Recently, the potential uses of Eugenol (EUG) have been considered a promising novel treatment for bacterial and parasitic infections. Consequently, it is suggested to investigate the effect of EUG as an option for the treatment of cryptosporidiosis.

**Materials and methods:**

The *in silico* bioinformatics analysis was used to predict and determine the binding affinities and intermolecular interactions of EUG and Nitazoxanide (NTZ) toward several *Cryptosporidium parvum* (*C. parvum*) lowa II target proteins. For animal study, five groups of immunosuppressed Swiss albino mice (10 mice each) were used. Group I was left uninfected (control), and four groups were infected with 1,000 oocysts of *Cryptosporidium* sp. The first infected group was left untreated. The remaining three infected groups received NTZ, EUG, and EUG + NTZ, respectively, on the 6th day post-infection (dpi). All mice were sacrificed 30 dpi. The efficacy of the used formulas was assessed by counting the number of *C. parvum* oocysts excreted in stool of infected mice, histopathological examination of the ileum and liver tissues and determination of the expression of iNOS in the ileum of mice in different animal groups.

**Results:**

treatment with EUG resulted in a significant reduction in the number of oocysts secreted in stool when compared to infected untreated mice. In addition, oocyst excretion was significantly reduced in mice received a combination therapy of EUG and NTZ when compared with those received NTZ alone. EUG succeeded in reverting the histopathological alterations induced by *Cryptosporidium* infection either alone or in combination with NTZ. Moreover, mice received EUG showed marked reduction of the expression of iNOS in ileal tissues.

**Conclusion:**

Based on the results, the present study signified a basis for utilizing EUG as an affordable, safe, and alternative therapy combined with NTZ in the management of cryptosporidiosis.

## Introduction

Cryptosporidiosis is a global opportunistic parasitic disease caused by the protozoan *Cryptosporidium* species (1). These parasites can infect mainly the epithelial cells of the jejunum and ileum of vertebrates after direct contact with the excreted oocysts in fecal materials or in the contaminated food, water, or drinks. Although, parasite development is relatively confined to the terminal jejunum and ileum, in immunosuppressed hosts the entire gastrointestinal tract as well as the biliary and pancreatic ducts may be infected and less frequently the respiratory tract (2).

Cryptosporidiosis may be self-limiting, or severe life-threatening condition depending on the immune status of the host (3–5). in immunocompetent individuals, *Cryptosporidium* infection may be a symptomatic or typically results in an episode of watery diarrhea (6). On the contrary, those with impaired immune systems such as infants, elderly and immunocompromised patients due to organ transplantation, AIDS and cancer therapy are more prone to infection with chronic, prolonged illness that is challenging to cure and may even be fatal (5, 7). In immunocompromised patients, cryptosporidiosis may cause severe life-threatening diarrhea and extra-intestinal disseminations resulting in bile duct obstruction, pancreatitis, papillary stenosis, and sclerosing cholangitis (5, 7, 8). Therefore, cryptosporidiosis is among the most serious opportunistic infections in immunocompromised patients (7).

Treatment options for cryptosporidiosis are extremely limited; there is no available vaccine is for this parasite, and nitazoxanide (NTZ) is the only FDA-approved drug for cryptosporidiosis. It promotes recovery in immunocompetent individuals but unfortunately has a very poor efficacy in children and in patients with acquired immunodeficiency syndrome (AIDS) (9). Treatment of the cause of immunosuppression has been found to reduce the severity of cryptosporidiosis in patients with Human immunodeficiency virus (HIV) and is not an option in immunocompromised patients without HIV infection (10, 11). Moreover, HIV patients in developing countries cannot afford anti-retrovirals, which results in the re-emergence of cryptosporidiosis (12).

Eugenol (EUG) (derived from the clove name, *Eugenia caryophyllata*) is the major phenolic constituent in several essential oils in clove, nutmeg, cinnamon, and basil (13). Due to its medicinal significance, EUG attracted the attention of researchers and created a vast field of study for its potential use as a medicine to treat a variety of disorders. Several pharmacological properties have been reported for EUG such as anesthetic, antioxidant, antibacterial, anti-helminthic, anti-inflammatory, anti-carcinogenic, schistosomicidal, anti-leishmanial, and anti-giardial properties (14–21).

Molecular docking is considered an important method that analyzes orientation of ligands into the binding sites of their targets. Searching algorithms generate poses that are ranked according to their scoring functions (22). For *Cryptosporidium*, the parasite depends mainly on glycolysis as a source of energy using LDH as a key for this process. So, this enzyme was used in many studies for assessment as a target protein for new therapeutics. In the present study we aim to assess the therapeutic potential of EUG in the treatment of cryptosporidiosis in immunocompromised mice and to use the *in-silico* bioinformatics analysis to predict and determine the binding affinities and many non-covalent intermolecular interactions of EUG and NTZ toward several *C. parvum* lowa II target proteins, including LDH, SerRS, TryptoRS, and MAPK1.

## Materials and methods

### *In silico* bioinformatics analysis

For ligands preparation, the PubChem database^1^ was used to obtain the canonical smiles of EUG (EUG, 2-methoxy-4-prop-2-enylphenol, MF:C_10_H_12_O_2_, MW:164.201 g/mol, CID: 3314) and NTZ [2-[(5-nitro-1,3-thiazol-2-yl)carbamoyl]phenyl] acetate, MF: C_12_H_9_N_3_O_5_S, MW: 307.28 g/mol, CID: 41684. Furthermore, ACD/ChemSketch program was used to generate, clean, and optimize the chemical structures of EUG and NTZ that were saved as MDL MOL-file formats. Moreover, OpenBabel GUI v2.3.2 software was used to minimize energy of the selected ligand compounds that converted from MDL MOL to PDB-file formats. For target proteins preparation, the crystal x-ray structures of *C. parvum* lowa II lactate dehydrogenase (LDH, PDB ID: 4ND1, 2.15 Å) (23, 24), cytoplasmic seryl-tRNA synthetase (SerRS, PDB ID: 6OTE, 2.95 Å), tryptophanyl-tRNA synthetase (TrpRS, PDB ID: 3HV0, 2.42 Å) (25), and mitogen-activated protein kinase1 (MAPK1, PDB ID: 3OZ6, 2.37 Å) were retrieved from the RCSB-PDB database^2^ as PDB-file formats. For energy minimization, the target proteins were processed using Swiss-PdbViewer v4.1.0 program.

Molecular docking was performed using Autodock v4.2.6 software that utilizes the estimated free energy of binding (kcal/mol) and inhibition constant (Ki) of EUG and NTZ toward their target proteins. For optimization, the structures of ligands were detected and chosen roots, which were saved as PDBQT-file formats. For protein optimization, water molecules, hetero atoms, and complex moieties were removed as well as polar hydrogen and Kollman and Gasteiger charges were added as PDBQT-file formats. For definition of the binding sites, the grid boxes were centered on macromolecules with 0.375 Å spacing, 18.017 X-, 2.772 Y-, and 28.774 Z-center, and 175 as several points in X-, Y-, and Z-dimensions. For the best docking conformation, Lamarckian Genetic Algorithm (GA) was applied in the drug-ligand interactions and 10 GA runs were performed with the following factors: 150 as population size, 250,000 as number of energy evaluations, and 27,000 as number of generations. The 10 conformations were clustered using a root-mean square deviation (RMSD) of 2.0 A. The least energy conformation was saved as a PDB-file format. If the binding energy is <−5 kJ/mol, it represents that the target protein has certain binding affinity toward the ligand (26–29). For the docked ligands, the elevated negative values of the estimated free energy of binding are positively correlated with their binding affinities and docking properties. For a better binding affinity, the binding energy should be lower (30). The pose with the best binding affinity was visualized using BIOVIA Discovery Studio Visualizer software.

### Animals and infection

The present study was carried out in the Parasitology department, Faculty of Medicine, South Valley University, Qena, Egypt. Fifty laboratory bred male Swiss albino mice weighing approximately 20 g were used. The mice had a 10-day acclimatization period before being infected with *Cryptosporidium* oocyst. All mice were kept in cages with proper ventilation, free supplies of water, and standard pellet food at a maintained temperature of 25°C with 12 h of light and 12 h of darkness. To exclude parasitic infections, stools were examined daily for 3 days.

All the experimental animals were subjected to immune suppression using dexamethasone orally at a dose of 0.25 mg/g/day for 14 successive days prior to inoculation with *Cryptosporidium* oocysts (31). The mice continued to receive dexamethasone at the same dose throughout the experiment (32–34).

Animals were divided into five groups, each with ten mice, as following: GI: immunocompromised non-infected mice; GII: immunocompromised *Cryptosporidium*-infected untreated; GIII: immunocompromised, *Cryptosporidium* infected and treated with NTZ; GIV: immunocompromised, *Cryptosporidium* infected and treated with EUG; GV: immunocompromised, *Cryptosporidium* infected and treated with NTZ + EUG. *Cryptosporidium* oocysts were kindly supplied by Theodor Belharz Institute, Cairo, EGYPT and were genetically identified by Dr. Eman El-Wakil as *C. parvum* (35). Mice in groups GII to GV were orally infected with 1,000 oocysts of *C. parvum* resuspended in 200 μL PBS for each mouse (36–38).

NTZ (Sigma Pharma, Egypt), was administered in a daily dose of 200 mg/kg (39, 40), while EUG, (Geno Technology Inco India, CAT #P8776-54), was administered at a dose of 500 μg/kg/day (20). All drugs were administered orally to the mice starting from the 6th dpi for five consecutive days. To confirm the establishment of infection, fresh fecal pellets were collected from each mouse on the 2nd dpi and examined using the modified Ziehl-Neelsen (mZN) staining method. All mice were euthanized on the 30th dpi and tissues (ileum and livers) were collected for evaluation of the efficacy of drugs.

### Assessment of infection and the drug efficacy

#### Stool examination

For evaluation of the efficacy of treatment, fresh fecal pellets were examined for each mouse on the 30^th^ dpi and examined using the mZN staining to determine the amount of *C. parvum* oocysts excreted on the last day of the experiment. Each sample was emulsified in 10% formal saline and 1 mg was smeared, fixed and stained with mZN, *C. parvum* oocysts were counted microscopically in 10 fields under 100x objective lens. The following formula was used to determine the percent reduction (PR), which represented the decline in the number of oocysts in the treated group compared to the infected untreated group.


$$\mathrm{Efficacy}\%=\;\frac{\begin{array}{l} \mathrm{mean}\;\mathrm{oocysts}\;\mathrm{count}\;\mathrm{in}\;\mathrm{control}\;\mathrm{group}-\\ \;\mathrm{mean}\;\mathrm{oocysts}\;\mathrm{count}\;\mathrm{in}\;\mathrm{treated}\;\mathrm{group} \end{array}}{\mathrm{mean}\;\mathrm{oocyst}\;\mathrm{count}\;\mathrm{in}\;\mathrm{control}\;\mathrm{group}}\times 100$$

### Histopathological examination

The last 2 cm of the ileum in addition to the liver were extracted from each euthanized animal, fixed in 10% formal saline, and processed into paraffin blocks. From each paraffin block, 3-mm thick portions were removed and stained with Hematoxylin & Eosin (H&E) by an independent pathologist (41). Intestinal tissue sections were examined for pathological findings and scored for the following: inflammation with villous atrophy (none = 0, slight = 1, moderate = 2, severe = 3); inflamed area/extent (mucosa = 1, mucosa and submucosa = 2, transmural = 3); surface ulceration (none = 0, focal = 1, diffuse = 2, complete loss of surface epithelium = 3, entire surface epithelium and crypt epithelium are lost = 4); percent involvement (1–25% = 1, 26–50% = 2, 51–75% = 3, 76–100% = 4) (42). Liver lesions were staged and graded according to the degree of periportal, periseptal interface hepatitis (piecemeal necrosis) (absent = 0, mild = 1, moderate = 2, severe = 3); confluent necrosis (absent = 0, focal = 1, zone 3 in some areas = 2, zone 3 in most areas = 3, zone 3 + occasional portal-central (P-C) bridging = 4, zone 3 + multiple P-C bridging = 5, panacinar or multiacinar = 6); focal (spotty) lytic necrosis, apoptosis and lobulitis per 10x objective (absent = 0, 1 focus = 1, 2–4 foci = 2, 5–10 foci = 3, > 10 foci = 4); portal lymphocytic inflammation (absent = 0, mild = 1, moderate = 2, marked = 3, strongly marked = 4).

### Immunohistochemical staining

The terminal 2 cm of the ileum were sectioned into 4-micron sections, incubated overnight, then deparaffinized, rehydrated in alcohol, and rinsed with dH_2_O. Endogenous peroxidase activity was blocked using 0.6% hydrogen peroxide (H_2_O_2_), then rinsed twice in PBS and boiled twice in Tris/EDTA buffer (pH 9.0). The rabbit recombinant monoclonal inducible nitric oxide synthase (iNOS) antibody was then applied to tissue sections and incubated for an additional overnight period at room temperature (Clone No., Abcam, Cambridge, MA, United States). After washing excess reagent in PBS containing 0.05% Tween-20 (PBS-T), tissue sections were incubated with HRP-conjugated goat anti-rabbit secondary antibody (1:5,000) (Vivantis Technologies, Selangor Darul Ehsan, Malaysia) at 4°C. After 1 h, slides were washed with PBS-T, and then incubated with 0.05% diaminobenzidine (DAB) and 0.01% H_2_O_2_ for 3 min to enhance the peroxidase reaction color. The smears were counterstained with hematoxylin for 1 min, then dehydrated, mounted, and then examined microscopically at different magnifications.

### Data analysis

The obtained data were analyzed using the Statistical Package for Social Sciences (SPSS) version 20 for Windows. All values were presented as mean ± standard deviation (SD). Analysis of Variance (ANOVA) followed by LSD *post hoc* analysis test was used for statistical comparison of different groups. *p*-value of < 0.05 was considered statistically significant.

## Results

### Evaluation of *in silico* bioinformatics findings

**Figure 1:**
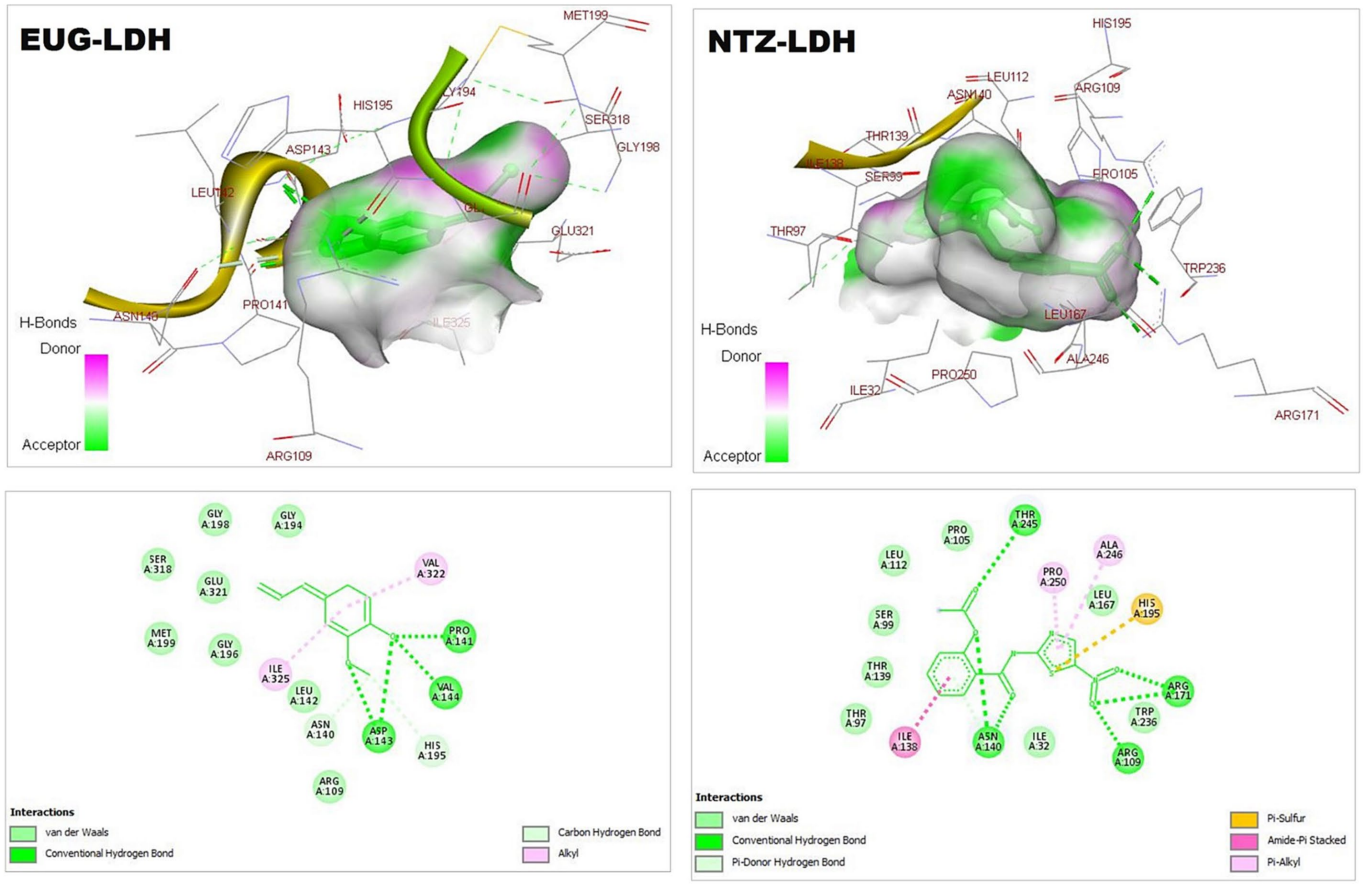
3D (upper panel) and 2D (lower panel) ligand-interacted forms show key amino acid residues of LDH using BIOVIA drug discovery studio visualizer. EUG, eugenol; NTZ, nitazoxanide; LDH, lactate dehydrogenase.

**Figure 2:**
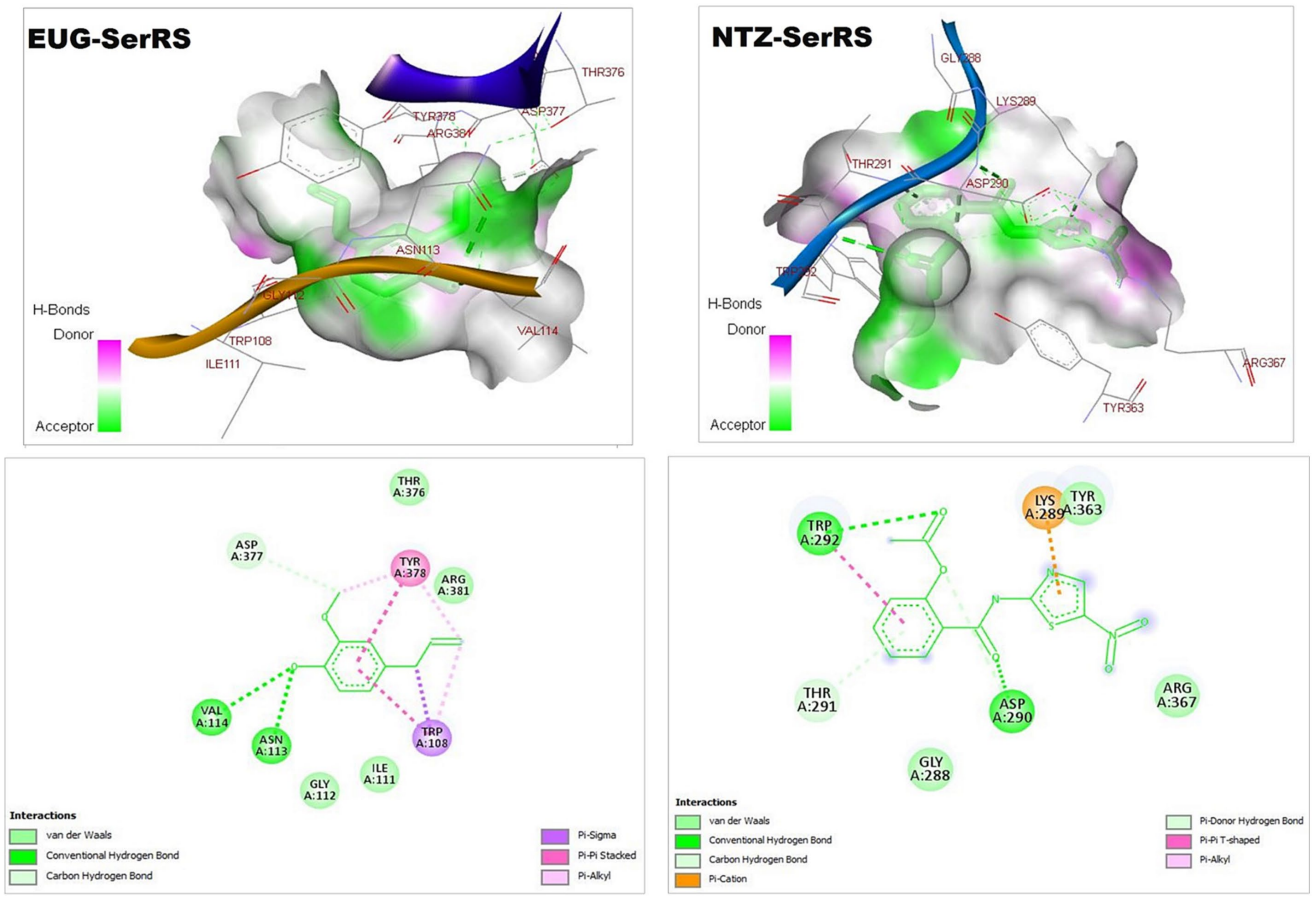
3D (upper panel) and 2D (lower panel) ligand-interacted forms show key amino acid residues of SerRS using BIOVIA drug discovery studio visualizer. EUG, eugenol; NTZ, nitazoxanide; SerRS, cytoplasmic seryl-tRNA synthetase.

**Figure 3:**
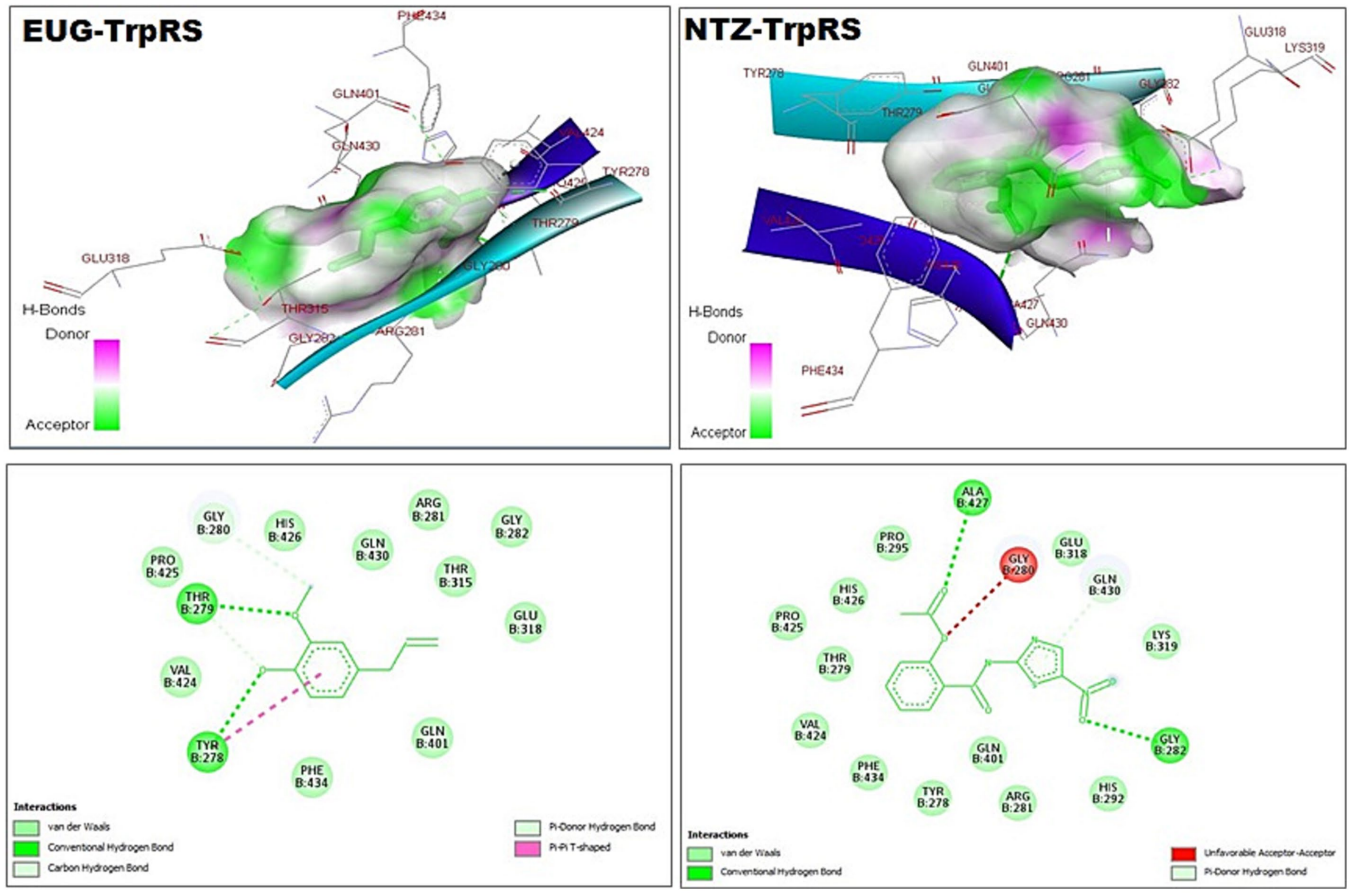
3D (upper panel) and 2D (lower panel) ligand-interacted forms show key amino acid residues of TrpRS using BIOVIA drug discovery studio visualizer. EUG, eugenol; NTZ, nitazoxanide; TrpRS, tryptophanyl-tRNA synthetase.

**Figure 4:**
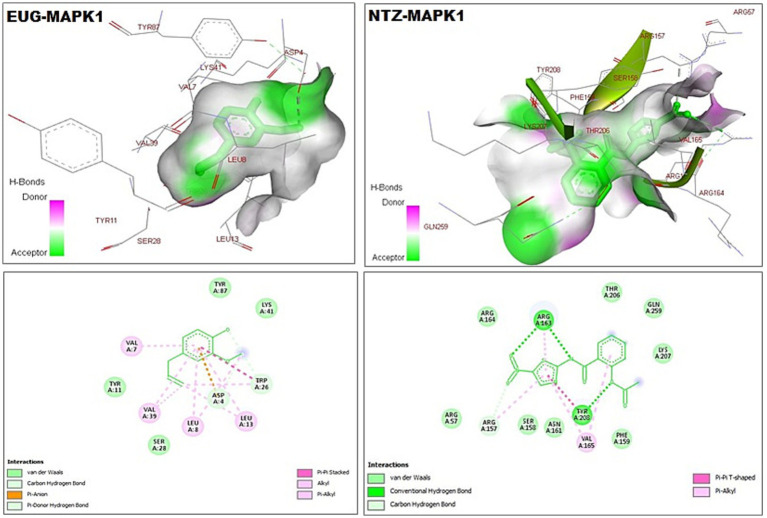
3D (upper panel) and 2D (lower panel) ligand-interacted forms show key amino acid residues of MAPK1 using BIOVIA drug discovery studio visualizer. EUG, eugenol; NTZ, nitazoxanide; MAPK1, mitogen-activated protein kinase1/serine–threonine protein kinase.

As reported in Table 1, the estimated free energy of binding of EUG toward *C. parvum* lowa II LDH, SerRS, TrpRS, and MAPK1 targets were −6.95, −6.51, −5.93, and −6.02 kcal/mol, respectively. While the estimated free energy of binding of NTZ toward *C. parvum* lowa II LDH, SerRS, TrpRS, and MAPK1 targets were −9.56, −7.41, −9.54, and −8.75 kcal/mol, respectively. Furthermore, the estimated inhibition constant values (Ki) of EUG toward LDH, SerRS, TrpRS, and MAPK1 targets were 8.08, 17.32, 44.64, and 39.51 μM, respectively. While the estimated Ki of NTZ toward LDH, SerRS, TrpRS, and MAPK1 targets were 97.69 nM, 3.71 μM, 102.43 nM, and 386.87 nM, respectively. Based on scoring functions, the strength of ligand-interacted forms is greatly associated to intermolecular binding and the interactions of the ligands and their target proteins such as classical/non-classical H-bond interaction, electrostatic interaction, and hydrophobic interaction (24), which confirmed our findings. As shown in Figures 1–4 and Table 1, the EUG and NTZ docked forms clearly demonstrated the different types, strength, and bond lengths of the intermolecular binding and interactions of EUG and NTZ toward their key amino acid residues of LDH, SerRS, TrpRS, and MAPK1 targets. Moreover, the NTZ-TrpRS docked form included an unfavorable acceptor-acceptor (Gly280B) interaction that could be reduced its stability compared to the EUG-TrpRS docked form (Figure 3; Table 1).

**Table 1:** The ligand-target protein binding properties.

Ligand-interacted properties			LDH (4ND1)	SerRS (6OTE)	TrpRS (3HV0)	MAPK1 (3OZ6)
EUG	Estimated free energy of binding (kcal/mol)		−6.95	−6.51	−5.93	−6.02
	Estimated inhibition constant (Ki)		8.08 μM	17.32 μM	44.64 μM	39.51 μM
	H-bonds	Amino Acids (Donor. Acceptor)	4 Conventional H-bonds (2 Asp143A (N…O), Val144A (N…O), Pro141A (O…O)); 2 carbon H-bonds (Asn140A (C…OD1), His195A (C…O))	2 Conventional H-bonds (Val114A (N…O), Asn113A (O…OD1)); carbon H-bonds (Asp377A (C…OD1))	2 Conventional H-bonds (Thr279B (OG1…O), Tyr278B (O…O)); 2 carbon H-bonds (Thr279B (*CA…*O), Gly280B (C…O)); Pi-Donor H-bond (Tyr278B (O-Pi))	Carbon H-bond (Asp4A (C…OD2)); Pi-Donor H-bond (Trp26A (O-Pi))
		Bond lengths (Å)	3.27, 3.21, 2.81, 2.81, 3.68, 2.67 Å	2.97, 3.22, 3.29 Å	2.65, 3.07, 2.97, 3.46, 4.18 Å	3.04, 3.83 Å
	Hydrophobic, miscellaneous, and electrostatic interactions		Alkyl hydrophobic Ile325A, Val322A	Pi-sigma, Pi-Pi stacked, and Pi-alkyl hydrophobic (Tyr378A, Trp108A)	Pi-Pi T-shaped hydrophobic (Tyr278B)	Pi-Pi Stacked, alkyl, and Pi-alkyl hydrophobic (Val7A, Val39A, Leu8A, Leu13A, Trp26A); electrostatic Pi-anion (Asp4A)
	Van der Waal’s reactions		Arg109A, Leu142A, Gly194A, Gly196A, Gly198A, Met199A, Ser318A, Glu321A	Thr376A, Arg381A, Gly112A, Ile111A	Pro425B, Val424B, His426B, Gln430B, Arg281B, Gly282B, Thr315B, Gln318B, Gln401B, Phe434B	Tyr87A, Tyr11A, Lys41A, Ser28A
NTZ	Estimated free energy of binding (kcal/mol)		−9.56	−7.41	−9.54	−8.75
	Estimated inhibition constant (Ki)		97.69 nM	3.71 μM	102.43 nM	386.87 nM
	H-bonds	Amino Acids (Donor. Acceptor)	6 Conventional H-bonds (Arg109A (NH2…O), 2 Asn140A (ND2…O), 2 Arg171A (NH1/NH2…O), Thr245A (OG1…O)); Pi-Donor H-bond (Asn140A (N…Pi orbitals))	2 Conventional H-bonds (Asp290A (N…O), Trp292A (N…O)); carbon H-bonds (Asp290A (*CA…*O)); 2 Pi-Donor H-bonds (Lys289A (NZ…Pi), Thr291A (N…Pi))	2 conventional H-bonds (Gly282B (N…O), Ala427B (N…O)); Pi-Donor H-bond (Gln430B (NE2…Pi))	3 Conventional H-bonds (Arg163A (NH1…O), Tyr208A (N…O), Arg163A (N…O)); carbon H-bond (Arg157A (CD…O))
		Bond lengths (Å)	3.08, 2.73, 2.87, 3.18, 2.85, 3.24, 3.65 Å	2.95, 2.64, 3.07, 3.53, 3.93 Å	2.92, 3.19, 3.88 Å	2.87, 2.83, 2.84, 2.97 Å
	Hydrophobic, miscellaneous, and electrostatic interactions		Miscellaneous Pi-sulfur (His195A); amide-Pi-stacked hydrophobic (Ile136A); Pi-alkyl hydrophobic (Pro250A, Ala246A)	Pi-Pi T-shaped and Pi-alkyl hydrophobic (Trp292A); electrostatic Pi-cation (Lys289A)	Unfavorable acceptor-acceptor (Gly280B)	Pi-Pi T-shaped and Pi-alkyl hydrophobic (Val165A, Tyr208A, Arg163A, Arg157A)
	Van der Waal’s reactions		Pro105A, Leu112A, Ser99A, Thr139A, Thr97A, Leu167A, Ile32A, Trp236A	Gly288A, Arg367A, Tyr363A	Pro295B, His426B, Pro425B, Thr279B, Val424b, Phe434B, Tyr278B, Gln401B, Arg281B, His292B, Glu318B, Lys319B	Arg164A, Arg57A, Ser158A, Asn161A, Phe159A, Thr206A, Gln259A, Lys207A

### *Cryptosporidium* oocysts count in different animal groups

**Figure 5:**
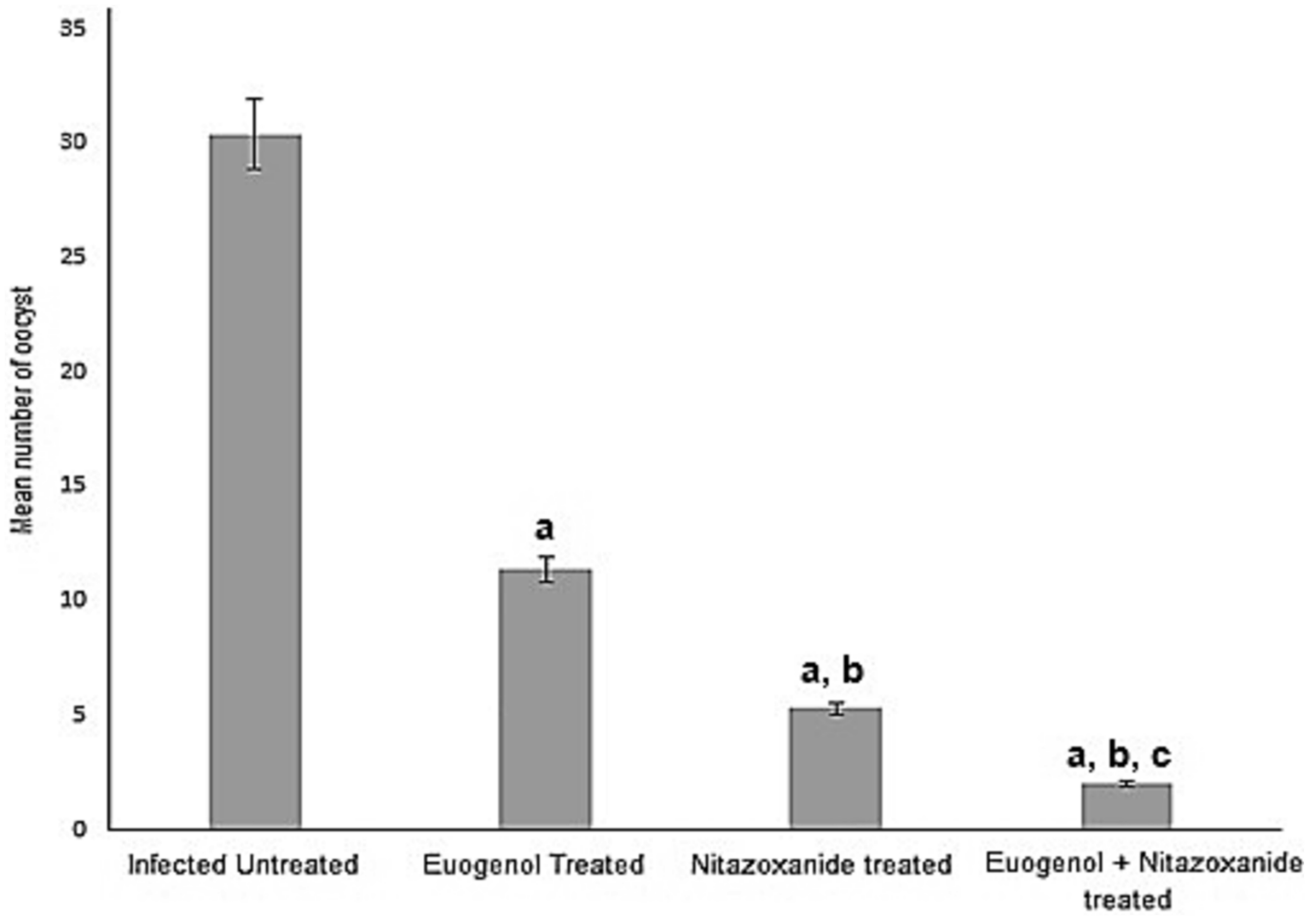
Treatment with eugenol reduced oocysts counts in the stool of infected mice. Oocysts were counted in infected untreated mice and compared to counts in animals treated with NTZ or EUG. Data are expressed as mean ± SD and were analyzed using ANOVA for pairwise comparison. Letter “a” indicates statistical difference compared to infected untreated group (*p* < 0.001). Letter “b” indicates statistical difference versus eugenol treated group (*p* < 0.001). Letter “c” indicates statistical difference versus NTZ treated group (*p* < 0.001).

The mean number of oocysts excreted in stool in all treated animals decreased significantly after treatment with EUG, NTZ, or EUG + NTZ when compared to the infected non-treated group shown in Table 2 and Figure 5 (*p* < 0.05). Additionally, when compared to NTZ alone, treatment with EUG and NTZ significantly reduced the amount of fecal oocyst (*p* = 0.02). animal groups treated with the combination of EUG and NTZ showed the highest percent reduction in the number of fecal oocyst (93.44%), followed by NTZ (82.56%) and EUG alone (62.5%; Table 2).

**Table 2:** Showing treatment with eugenol reduced oocysts counts in the stool of infected mice.

Animal group	Oocyst count/HPF Mean ± SD	*%R*	*p* value (among groups)	post hock test
Infected untreated	30.4 + 3.33		0.001*	
Infected + Eugenol	11.4 + 1.33	62.5%		a, b
Infected + NTZ	5.2 + 0.6	82.56%		a
Infected + Eugenol + NTZ	1.9 + 0.2	93.44%		a, c

Oocysts were counted in infected untreated mice and compared to counts in animals treated with NTZ or euogenol or both. Data are expressed as mean ± SD and were analyzed using ANOVA for pairwise comparison.

%R: percentage of reduction. SD: Standard deviation. * indicates statistical significance. Letter “a” indicates significant reductions in oocysts counts after treatments compared to infected untreated group. Letter “b” indicates a significant difference in oocysts versus NTZ treated group. Letter “c” indicates a significant difference in oocysts versus eugenol treated group.

### Histopathology of the small intestine

**Figure 6:**
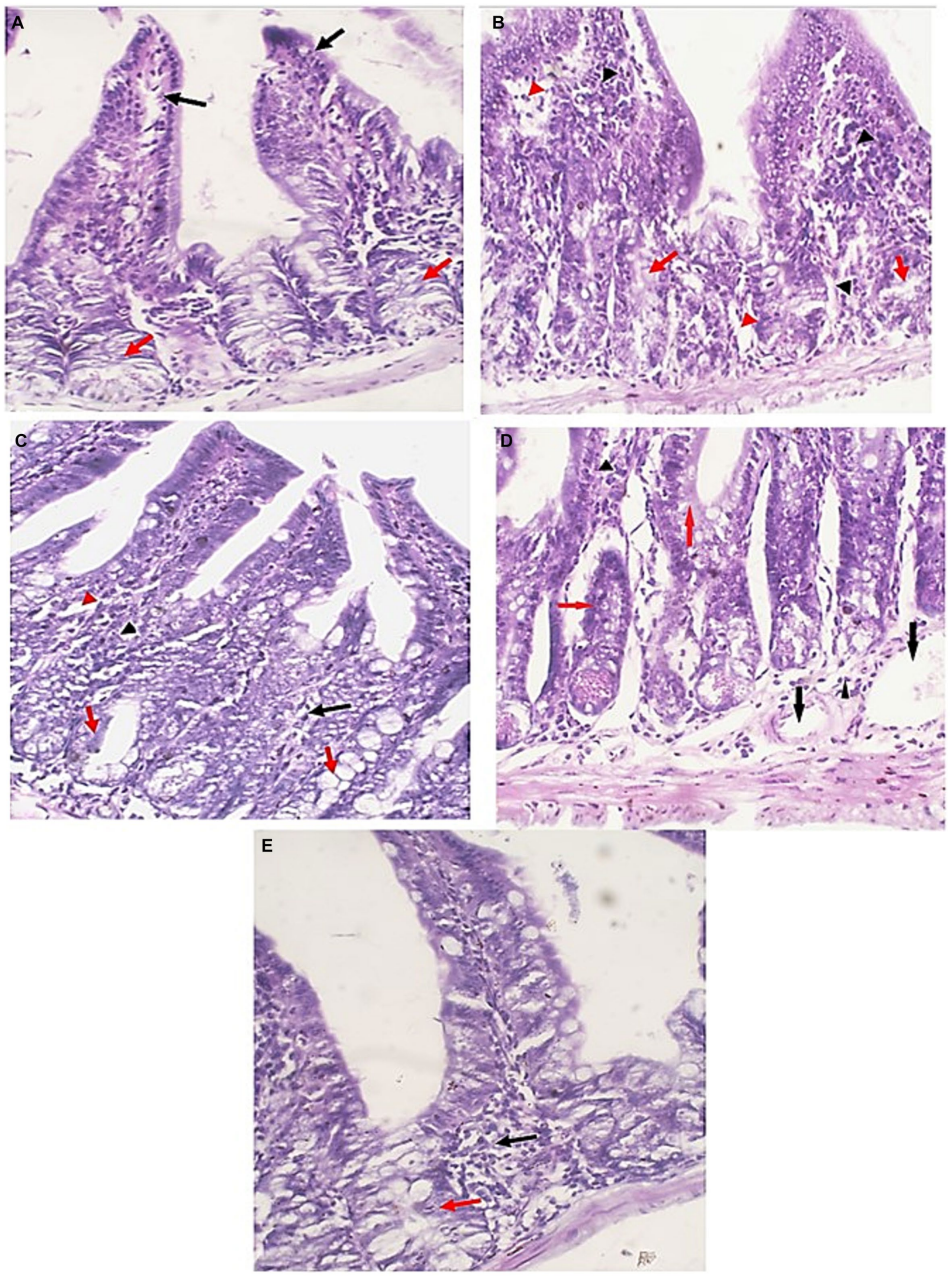
Small intestine sections of all studied groups. **(A)** Sections of uninfected untreated mice showing uniform intestinal tissue showing regular villi (Black arrows) with uniform crypts and glands in the lamina propria (Red arrows) (H&E, 400×). **(B)** Sections of infected untreated mice showing expansion of the lamina propria with chronic inflammatory cells (Black arrowheads). Glands (Red arrows) are distorted and attacked by chronic inflammatory cells. There are areas of edema (Red arrowheads) (H&E, 400×). **(C)** Sections of infected EUG treated mice showing significant reduction in the severity and the extent of inflammation (Black arrowheads) and edema (Red arrowheads) in the mucosa. Glands appear more uniform with no inflammatory cells attacking the glands (Red arrows) (H&E, 400×). **(D)** Sections of infected NTZ treated mice showing restoration of intestinal tissue, with mild chronic inflammatory cells infiltrate in the lamina propria (Black arrowheads). Glands retained their uniform regular outlines (Red arrows) with dilated blood vessels (Blue arrows) (H&E, 400×). **(E)** Sections of infected NTZ+ EUG treated mice showing restoration of intestinal tissue, with mild chronic inflammatory cells infiltrate in the lamina propria (Black arrowheads). Glands show uniform regular outlines (Red arrows) (H&E, 400×).

In contrast to uninfected mice, sections of ileum of infected untreated group (GI) showed villous blunting with moderate transmural inflammatory cellular infiltration (mainly lymphocytes) involving more than 75% of the intestinal wall with marked reduction of goblet cells. Treatment of mice caused significant restoration of normal epithelial structure, restoration of goblet cells and lowered the inflammatory infiltration of the intestinal wall. EUG alone resulted in marked reduction of cellular infiltration, which was restricted to the mucosa only in less than 30% of the intestinal wall. Similar results were recognized in mice treated with NTZ where there was almost restoration of intestinal tissue, with mild chronic inflammatory cells infiltrate in the lamina propria with some dilated blood vessels. The best degree of improvement was recognized in the mice group treated with NTZ and EUG combination (Figure 6).

**Figure 7:**
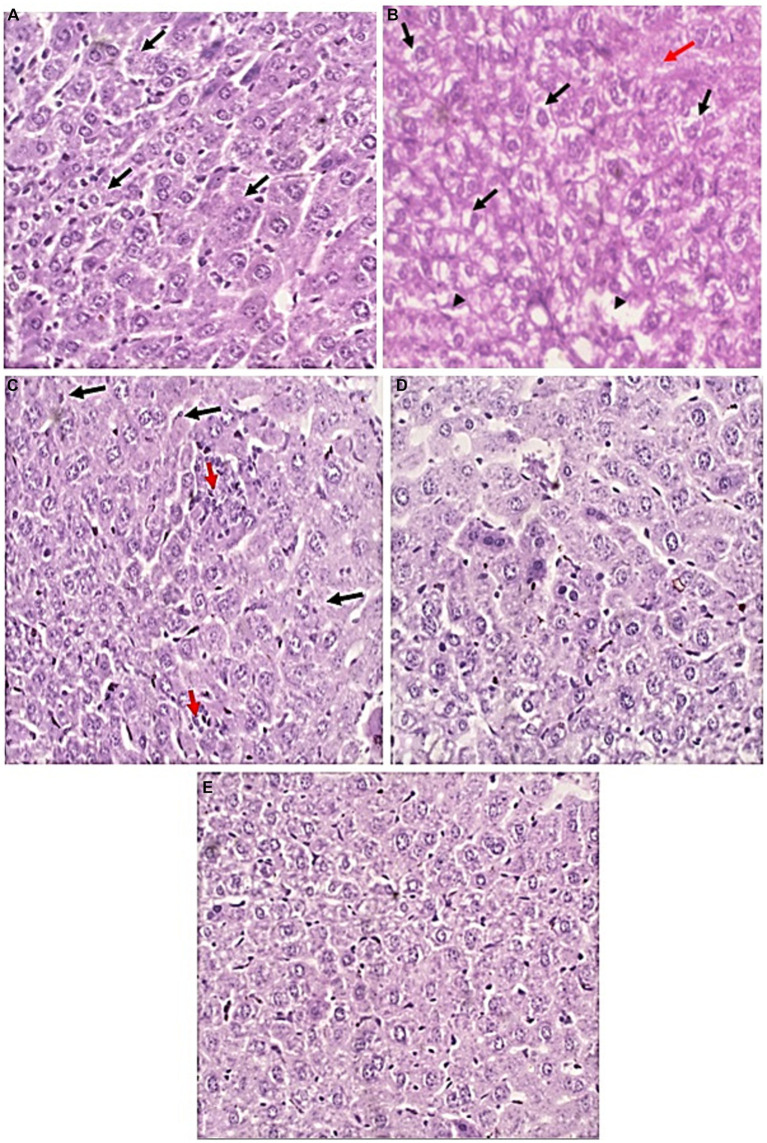
Liver sections of all studied groups. **(A)** Sections of uninfected untreated mice showing Uniform plates of hepatocytes (Black arrows) with no evidence of injury (H&E, 400×). **(B)** sections of infected untreated mice showing significant hydropic degeneration of hepatocytes (Black arrows). There are areas of lytic necrosis (Black arrowheads) with confluent necrosis (Red arrow) (H&E, 400×). **(C)** Sections of infected EUG treated mice showing Hepatocytes show no evidence of hydropic degeneration (Black arrows). There are two foci of lobulitis (Red arrow) (H&E, 400×) **(D)** Sections of infected NTZ treated mice showing restoration of hepatocytes integrity with no evidence of injury (H&E, 400×). **(E)** sections of infected NTZ+ EUG treated mice showing restoration of hepatocytes integrity with No evidence of injury.

### Histopathology of the liver

H&E-stained liver sections were used to examine the therapeutic effect of EUG in the alleviation of pathological changes in the liver of immunocompromised mice with cryptosporidiosis. Liver tissues of uninfected mice showed normal uniform plates of hepatocytes (Black arrows) with no evidence of injury. Liver tissues of infected untreated mice showed significant hydropic degeneration of hepatocytes (Black arrows), lytic necrosis (Black arrowheads) and confluent necrosis (Red arrow). Treated groups showed marked improvement in comparison to uninfected ones. In EUG treated mice, hepatocytes showed mild lobulitis (Red arrow) with no evidence of hydropic degeneration (Black arrows). On the other hand, mice treated with NTZ showed restoration of hepatocytes integrity with no evidence of injury. Combination of NTZ and EUG showed restoration of hepatocytes integrity with no evidence of injury (Figure 7).

### Immunohistochemistry

**Figure 8:**
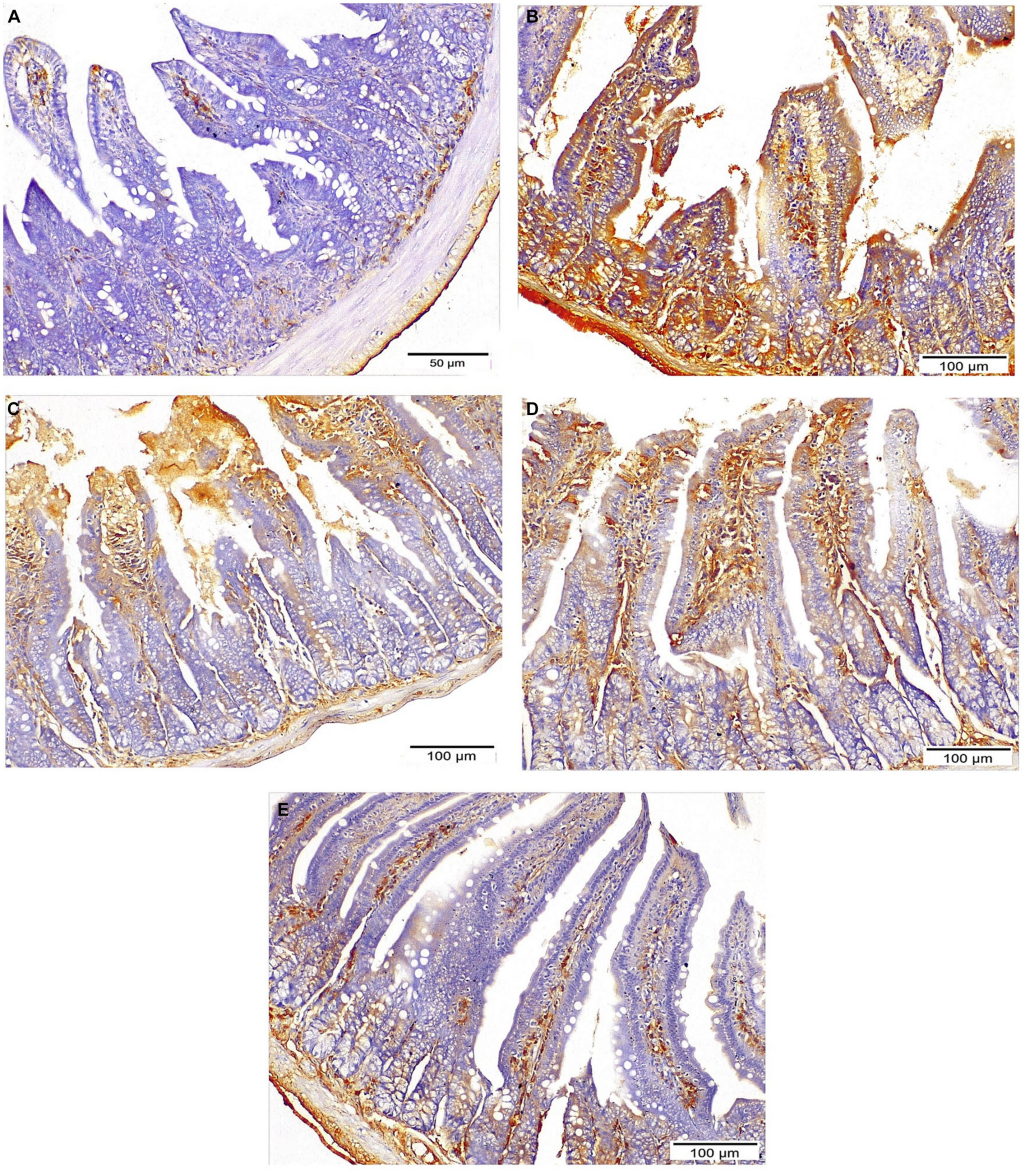
Sections of ileum showing INOS expression: **(A)** Negative expression in normal control mice. **(B)** Strong expression in infected untreated group (IHC ×200). **(C,D)** Moderate expression in both NTZ and EUG treated groups, respectively, (IHC ×200). **(E)** Mild diffuse expression in NTZ + EUG treated GROUP (IHC ×200).

Strong cytoplasmic expression of iNOS were detected in the intestinal epithelium of the infected untreated mice, while the treated groups revealed weaker expression with significant reduction of the mean percent of positive expression (*p* = 0.002). Mice that received combined treatment (NTZ + EUG) showed the lowest mean percentage of iNOS positive cells (Table 3 and Figure 8).

**Table 3:** Showing the percentage of iNOS positive cells in ileal tissues of different animal groups.

Animal group	% of positive cells Mean ± SD	*p* value (among groups)	post hock test
Uninfected untreated	2.67 ± 1.2		
Infected untreated	40.01 ± 1.9	0.002*	a*
Infected + NTZ	23.2 ± 2.2		a*, b*
Infected + Eugenol	28.6 ± 2.1		a*, b*
Infected + Eugenol + NTZ	16.04 ± 2.5		a*, b*, c*

Data are expressed as mean ± SD and were analyzed using ANOVA for pairwise comparison. Letter “a” indicates statistical difference compared to infected untreated group (*p* < 0.001). Letter “b” indicates statistical difference versus eugenol treated group (*p* < 0.001). Letter “c” indicates statistical difference versus NTZ treated group (*p* < 0.001).

## Discussion

Bioinformatics is a potent biological area that uses computational-based methods to evaluate the biological systems and provide some accurate predictions for several *in vitro* and *in vivo* studies and clinical trials (24, 43). The molecular docking method determines ligand conformation and orientation within a targeted binding site. Searching through algorithms generates conformations that are ranked according to their scoring functions (22, 24). Plant essential oils potentially have ecotoxicological activities against several parasites and insects (44). EUG (4-allyl-2-methoxyphenol) is considered as a phenylpropanoid compound that represents a major component of plant essential oils and has an allyl chain-substituted guaiacol. Naturally, EUG is present in many plant families and several aromatic plants (45, 46). Previous studies reported that EUG has potent medicinal therapeutic applications including antimicrobial, antiviral, antiparasitic, anti-inflammatory, neuroprotective, antioxidant, anti-diabetic, anti-obesity, hypolipidemic, and anticancer potentials (47–49). In the present study, we targeted molecular docking simulation analysis to visualize, determine, and evaluate the binding affinities and inhibition potentials of EUG and NTZ against some *C. parvum* lowa II target proteins including LDH, SerRS, TrpRS, and MAPK1. In this study, the lower docking scores of the EUG- and NTZ-interacted forms represented strength of the ligand-target protein binding activities, stability of the binding conformation, and variety of the intermolecular binding and interactions (Figures 1–4; Table 1), which confirmed recent findings (50).

*Cryptosporidium* is a protozoan parasite that potentially causes waterborne diseases. The parasite depends on glycolysis for energy production and cellular metabolism. *C. parvum* LDH is an essential regulator of glycolysis. The anti-cryptosporidial drugs aim to target and inhibit the biochemical and metabolic pathways of *C. parvum* (23). As critical substrate-binding sites, Arg171, Asn140, His195, Arg109, and Trp236 represent the catalytic amino acid residues of *C. parvum* LDH (23), which clearly presented in the EUG-LDH and NTZ-LDH docked forms (Figure 1).

*C. parvum* lowa II SerRS and TrpRS are considered as important protozoan enzymes that widely regulate protein biosynthesis (25). As shown in Figures 2, 3 and Table 1, the EUG and NTZ docked forms potentially demonstrated the binding affinities, inhibition potentials, and the intermolecular interactions of EUG and NTZ toward amino acid residues of *C. parvum* lowa II SerRS and TrpRS targets, which greatly predicted inhibition of differentiation, growth, and survival of *C. parvum* cells. Merritt et al. study reported that Glu318, Gln401, Gly280, Thr279, and Tyr278 as key amino acid residues represented the active site of *C. parvum* TrpRS (25), which clearly demonstrated in the EUG- and NTZ-interacted forms (Figure 3; Table 1). Moreover, the NTZ-TrpRS docked form had an unfavorable acceptor-acceptor (Gly280B) interaction that have reduced its stability compared to the EUG-TrpRS docked form.

MAPK is a serine/threonine protein kinase that regulates cellular growth, development, differentiation, survival, and interaction interactions between host and various pathogens, including parasites (51–53). In lung injury, inhibition of the MAPK3/MAPK1 signaling process highly reduces cellular inflammation, oxidative stress response, pro-inflammatory cytokines, and apoptotic signaling (50, 54, 55). This study reported that EUG and NTZ were introduced as *C. parvum* MAPK1 inhibitors that greatly confirmed findings of previous studies.

Immunosuppression may arise as a side effect of cancer treatment, in HIV-positive people, or following organ donation. Consequently, this category of patients are more prone to opportunistic infection including *Cryptosporidium* species with high probability of severe life-threatening illnesses especially with limited treatment options (56, 57). Some efforts have been focused on evaluating natural compounds, particularly essential oils against *C. parvum* (58). Therefore, the purpose of the present study was to assess the effectiveness of EUG in treating immunosuppressed mice infected with *C. parvum*. For the induction of a mouse model of immunosuppression, dexamethasone was used. Dexamethasone was used in the present study for induction of immune suppression following previous studies (31, 33, 34, 59, 60). Convincing evidence shows that EUG possesses potent antimicrobial, antifungal, antibacterial, and anti-parasitic properties (14–21), however, information about its anti-cryptosporidial properties is limited. So, we aimed to evaluate the efficacy of EUG against *C. parvum in vivo*. Our research revealed that EUG effectively combats *C. parvum* in immunocompromised mice. To the best of our knowledge, this is the first study that evaluates Euogenol anti-cryptosporidial activity *in vivo* in an immunocompromised mice model. Our findings show that EUG has considerable anti-cryptosporidial action and a significant synergistic effect when combined with NTZ. Comparing treated and untreated mice, the current study found that EUG considerably reduced the degree of oocyst shedding. Additionally, when combined with NTZ, EUG significantly reduced the number of oocysts that were shed, as opposed to NTZ alone, which, according to earlier studies, was unable to entirely remove the oocysts (34).

Tasdemir et al. conducted an *in vitro* analysis of EUG’s effectiveness against *Cryptosporidium* oocysts (61). According to their findings, thyme, oregano, and clove essential oils can significantly reduce the quantity of *Cryptosporidium* oocysts.

To further evaluate the effect of EUG on *C. parvum* induced pathological changes in the intestine and liver tissues, H & E-stained sections of both tissues were examined. Small intestine sections of animals infected with the *C. parvum* and subsequently treated with EUG either alone or in combination with NTZ showed a normal villous pattern with a mild lymphocytic inflammatory response noted in the villi and lamina propria. In the same line, EUG treatment-either alone or in combination with NTZ-restored normal liver histological structures and alleviated *Cryptosporidium* induced alterations.

Furthermore, immunohistochemical screening of iNOS antibody revealed strong cytoplasmic expression in the intestinal epithelium of infected untreated mice. In contrast, weak expression was observed in mice treated with EUG alone or in combination with NTZ. This confirmed the strong oxidative stress strived by the parasite and confirmed the effect of drugs in reducing oxidative stress in tissue. This result agrees with previous studies which demonstrated that mice and piglets infected with *Cryptosporidium*, were able to recover after treatment with iNOS inhibitor or peroxynitrite scavenger, suggesting that reactive nitrogen intermediates may serve as an early and innate defense against intestinal epithelial infection (62). It was documented that the synthesis of NO is increased in cryptosporidiosis, while the inhibition or absence of iNOS decreased epithelial infection and oocyst shedding (63).

The mechanisms behind the antiprotozoal effect of essential oils need further investigations to be fully understood. The high lipophilic nature of the essential oils allows easy absorption by the cell membrane and inhibition of the lipid metabolism of parasites. Another mode of action involves penetrating the membrane first, followed by modulation of cytoplasmic metabolic pathways or organelle function, rather than compromising the integrity of the parasite’s membrane (64).

## Conclusion

*Cryptosporidium* is an opportunistic causing with life threatening illness in immunocompromised individuals with limited treatment options. In the present study EUG was able to combat cryptosporidiosis alone and had a synergistic effect when added to NTZ. Mice received EUG alone or in combination with NTZ showed reduced fecal oocyst count and restored the normal histological structures of the liver and spleen when compared with non-treated mice. Based on the results, the present study signified a basis for utilizing EUG as an affordable, safe, and alternative therapy combined with NTZ in the management of cryptosporidiosis.

## Data availability statement

The original contributions presented in the study are included in the article/supplementary material, further inquiries can be directed to the corresponding authors.

## Ethics statement

The animal study was approved by the Faculty of Medicine’s Institutional Review Board and Ethics Committee at South Valley University, Qena, Egypt (SVU-MED-PAR008-4-22-9-436). The study was conducted in accordance with the local legislation and institutional requirements.

## Author contributions

HG: Writing – review & editing. MW: Writing – review & editing. RQ: Writing – review & editing. SA: Writing – review & editing. HM: Writing – review & editing. MA: Writing – review & editing. HayE: Writing – original draft. WA-M: Funding acquisition, Writing – review & editing. EA: Writing – review & editing. HatE: Writing – original draft, Writing – review & editing. AE-k: Conceptualization, Data curation, Formal analysis, Funding acquisition, Investigation, Methodology, Project administration, Resources, Software, Supervision, Validation, Visualization, Writing – original draft, Writing – review & editing.

## Footnotes

^1^ https://pubchem.ncbi.nlm.nih.gov/

^2^ http://www.rcsb.org/
