# Corrigendum: *In silico* and *in vivo* evaluation of the anti-cryptosporidial activity of eugenol

**DOI:** 10.3389/fvets.2024.1407833

**Published:** 2024-05-08

**Authors:** Hattan S. Gattan, Majed H. Wakid, Rowaid M. Qahwaji, Sarah Altwaim, Haifaa A. Mahjoub, Mashael S. Alfaifi, Hayam Elshazly, Wafa Abdullah I. Al-Megrin, Eman Abdullah Alshehri, Hatem A. Elshabrawy, Asmaa M. El-kady

**Affiliations:** ^1^Department of Medical Laboratory Sciences, Faculty of Applied Medical Sciences, King Abdulaziz University, Jeddah, Saudi Arabia; ^2^Special Infectious Agents Unit, King Fahd Medical Research Center, Jeddah, Saudi Arabia; ^3^Department of Clinical Microbiology and Immunology, Faculty of Medicine, King Abdulaziz University, Jeddah, Saudi Arabia; ^4^Biological Sciences Department, College of Sciences and Arts, King Abdulaziz University, Rabigh, Saudi Arabia; ^5^Department of Epidemiology, Faculty of Public Health and Health Informatics, Umm Al-Qura University, Mecca, Saudi Arabia; ^6^Department of Biology, Faculty of Sciences-Scientific Departments, Qassim University, Buraidah, Saudi Arabia; ^7^Department of Zoology, Faculty of Science, Beni-Suef University, Beni Suef, Egypt; ^8^Department of Biology, College of Science, Princess Nourah Bint Abdulrahman University, Riyadh, Saudi Arabia; ^9^Department of Zoology, College of Science, King Saud University, Riyadh, Saudi Arabia; ^10^Department of Molecular and Cellular Biology, College of Osteopathic Medicine, Sam Houston State University, Conroe, TX, United States; ^11^Department of Medical Parasitology, Faculty of Medicine, South Valley University, Qena, Egypt

**Keywords:** *Cryptosporidium*, immunocompromised, eugenol, nitazoxanide, iNOS

In the published article, there was an error in the Funding statement. The funding universities request to add this exact sentence with no changes to grant numbers, only rephrasing the paragraph. This correction is required since this error is going to affect awards disposition at this University.

The correct Funding statement appears below.

